# Presence of the *HPPD Inhibitor Sensitive 1* Gene and ALS^S653N^ Mutation in Weedy *Oryza sativa* Sensitive to Benzobicyclon

**DOI:** 10.3390/plants9111576

**Published:** 2020-11-14

**Authors:** Chad Brabham, Jason K. Norsworthy, Fidel González-Torralva

**Affiliations:** Department of Crop, Soil, and Environmental Sciences, University of Arkansas, Fayetteville, AR 72704, USA; chad.brabham@fmc.com (C.B.); jnorswor@uark.edu (J.K.N.)

**Keywords:** cultivated rice, *japonica*, *indica*, *aus*, IMI-resistant

## Abstract

Benzobicyclon has shown varying results in controlling weedy rice, including those with imidazolinone (IMI) resistance. Tolerance to benzobicyclon in cultivated *japonica* rice, but not *indica* or *aus*-like cultivars, is conferred by a fully functional *HPPD Inhibitor Sensitive 1* (*HIS1*) gene. Herein, a diagnostic Kompetitive Allele Specific PCR (KASP) assay was developed to predict the *HIS1* genotype of weedy rice plants from 37 accessions and correlated to their response to benzobicyclon in the field. Two-thirds of the 693 weedy rice plants screened were tolerant to benzobicyclon (371 g ai ha^−1^, SC formulation) at 30 days after treatment (DAT). Thirty-four percent of plants were homozygous for the *HIS1* allele and 98% of these plants exhibited field tolerance. However, the *his1* genotype did not always correlate with field data. Only 52% of *his1* plants were considered sensitive, indicating that the single nucleotide polymorphisms (SNPs) chosen in the KASP assay are not a reliable tool in predicting *his1* homozygous plants. In an additional experiment, 86% of the 344 plants with at least one copy of the ALS^S653N^ trait harbored a *HIS1* allele, suggesting fields infested with IMI herbicide-resistant weedy rice are unlikely to be controlled with benzobicyclon.

## 1. Introduction

In rice (*Oryza sativa* L.) fields worldwide, weedy rice is a troublesome weed that can cause economic losses by reducing grain yield and/or grain quality [1,2]. Season-long interference of weedy rice at 2 or 40 plants m^−2^ was found to reduce the grain yield of different cultivars by 7 to 19% and 61 to 87%, respectively [3,4]. Taxonomically, weedy rice and cultivated rice are the same species, and thus selective chemical control of weedy rice in cultivated rice is extremely difficult. Cultural practices such as crop rotation, stale seedbed, water seedings, or transplanting instead of direct-seeding are some of the methods used around the world to deter weedy rice infestations [5]. In the 2000s, non-transgenic IMI-resistant (Clearfield^TM^ technology, BASF) rice became commercially available in the midsouthern United States and served as an effective tool for controlling many weeds of rice, including weedy rice. IMI-resistant cultivars have a mutation in the acetolactate synthase (*ALS*) gene. The original Clearfield lines contained a mutated ALS^G654E^ gene, but were quickly replaced by cultivars harboring the ALS^S653N^ trait because of their superior yield and improved tolerance to IMIs [2,6,7]. Unfortunately, owing to the natural gene flow between IMI-resistant rice and weedy rice in conjunction with the overreliance of IMIs, IMI-resistant weedy rice accessions quickly appeared and are now prevalent throughout the rice growing regions in the midsouthern United States [8].

The pro-herbicide benzobicyclon is a 4-hydroxyphenylpyruvate dioxygenase (HPPD)-inhibitor under registration in the midsouthern United States for control of several annual grasses, sedges, broadleaves, and aquatic weeds in flooded rice [9,10]. Rice sensitivity to benzobicyclon is cultivar-specific, but at broad-scale, tolerance diverges among rice subspecies. The two major cultivated rice subspecies worldwide are *japonica* and *indica*, where *japonica* cultivars are typically tolerant, while *indica* cultivars are highly sensitive [10,11]. In *japonica* cultivars, tolerance to benzobicyclon is conferred by a fully-functional *HIS1* gene found on chromosome two in the rice genome. *HIS1* belongs to the Fe(II)/2OG-dependent oxygenase family, similar to the HPPD enzyme, and was found to hydroxylate benzobicyclon hydrolysate and not benzobicyclon into a less phytotoxic compound [12]. The *his1* allele in *indica* cultivars contains a 28 bp deletion loss-of-function mutation that results in an early stop codon and encodes for a severely truncated protein [12]. Additionally, the *HIS1* gene in *aus* rice cultivars such as Kasalath [13] and Purple Marker [10] do not contain a 28 bp deletion, yet *aus* cultivars appear to be sensitive to benzobicyclon and will hereafter be referred to as HIS1 *. In comparison with the *HIS1* gene in *japonica* cultivars, several mutations can be found near the 3′ end of the HIS1 * gene in *aus* cultivars and may alter protein function, but this has not been proven.

Weedy rice in the midsouthern United States is morphologically and phenotypically diverse with two suggested groups based on seed characteristics: strawhull (awnless) and blackhull (awned) [14]. Genetic analysis by Shivrain et al. [15] indicated weedy rice arose from hybridizations events between *O. rufipogon, O. sativa indica,* and *aus*, while Reagon et al. did not find unambiguous evidence for genetic contribution from *O. rufipogon* [16]. However, significant admixture has occurred between weedy rice and the popular *japonica* cultivars grown in the midsouth (e.g., Clearfield-resistant weedy rice). Interestingly, in benzobicyclon-containing research plots in Arkansas, benzobicyclon was serendipitously found to have significant activity on weedy rice [17]. This prompted Young and co-authors [10] to access the sensitivity of weedy rice accessions collected across the midsouthern United States to benzobicyclon. The authors found the response of weedy rice plants to benzobicyclon at 371 g ha^−1^ (SC formulation) varied considerably within and among accessions. In addition, they found that, in the field, 30 out of the 100 accessions screened exhibited at least 80% control (~50 plants plot area^−1^).

In the current study, the benzobicyclon sensitivity of 37 weedy rice accessions used by Young et al. [10] was further dissected. Specifically, 14 to 24 weedy rice plants per accession were subjected to benzobicyclon in the field and later genotyped for the *HIS1*/*his1* alleles and for the ALS^S653N^ trait using KASP. KASP is a common quantitative PCR genotyping technique designed to identify allele specific SNPs [18]. The overall objective of this study was to develop diagnostic tools to predict the effectiveness of benzobicyclon in a growers’ weedy rice management program.

## 2. Results and Discussions

### 2.1. Sensitivity of Weedy Rice to Benzobicyclon

The percent control of 37 different weedy rice accessions (14 to 24 plants per accession) when treated with benzobicyclon (371 g ha^−1^) at 30 DAT averaged 28% (Table S1). Based on the response of all plants within an accession, 21, 8, and 8 of the accessions tested were classified (>90% of plants) as benzobicyclon tolerant, mixed, or susceptible, respectively. On a plant basis, 69% of the 693 plants screened were classified as benzobicyclon tolerant. Of the remaining 216 sensitive plants, 77% were killed, with the rest significantly injured (>50% injury) at 30 DAT. These results are consistent with Young and co-authors [10], who found benzobicyclon did not adequately (<80%) control 70 out of the 100 accessions screened and noted several accessions were a heterogenous mixture of sensitive and tolerant weedy rice plants.

### 2.2. HIS1 Genotype of Weedy Rice Plants

**Figure 1:**
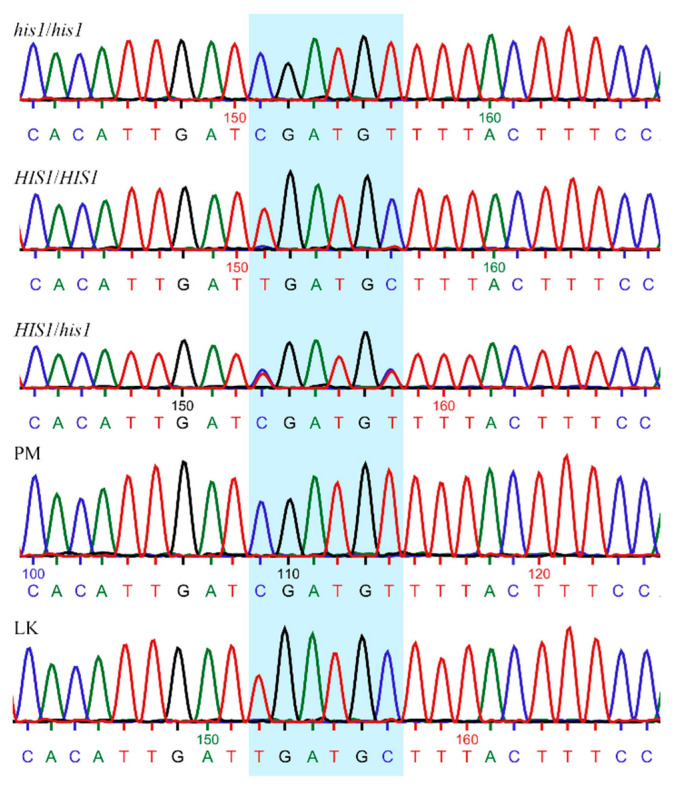
Chromatograms of Sanger sequencing showing partial *HIS1* sequence and the target used (highlighted) for genotyping the different weedy rice accessions. Rice varieties known for their susceptibility (PM) and tolerance to benzobicyclon (LaKast) were used as reference. *his1*/*his1*: homozygous susceptible; *HIS1*/*HIS1*: homozygous resistant; *HIS1*/*his1*: heterozygous; PM: Purple Marker; LK: LaKast.

The 693 plants from the field assay were genotyped for the *HIS1* gene using a KASP assay. The assay was designed to distinguish between the *HIS1* allele found in *japonica* rice and the *HIS1* */*his1* alleles found in sensitive *aus*/*indica* cultivars. Although plants harboring the *HIS1* * or *his1* alleles have differing levels of sensitivity to benzobicyclon [13], both allelic forms in this assay are considered sensitive and cannot be differentiated. *HIS1* */*his1* will hereafter be referred to as *his1*. Sanger sequencing results agreed with the KASP assay calls, which highlights the reliability of our assay to differentiate between *HIS1* and *his1* alleles based on the chosen SNPs (Figure 1).

To determine a typical response of weedy rice plants within an accession, we broadly classified accessions based on their *HIS1* allele frequency; where an accession was considered tolerant if ≥90% of the alleles were *HIS1*, mixed if 10% < *HIS1* < 90%, or sensitive if *HIS1* ≤ 10%. Out of the 37 accessions screened, 7, 15, and 15 were predicted to be a tolerant, mixed, or sensitive population, respectively (Table 1 and Table S1). All seven accessions classified as tolerant in the KASP assay were classified as tolerant in the field experiment and, furthermore, on a plant level, 98% of all homozygous *HIS1* plants were biologically tolerant to benzobicyclon. However, the genotypic classification of an accession was not always analogous with the field results. In fact, only 18 out of the 37 accessions tested had similar classifications. These results indicate that the SNPs chosen in the KASP assay are not a reliable tool in predicting *his1* homozygous plants’ sensitivity to benzobicyclon.

**Figure 2:**
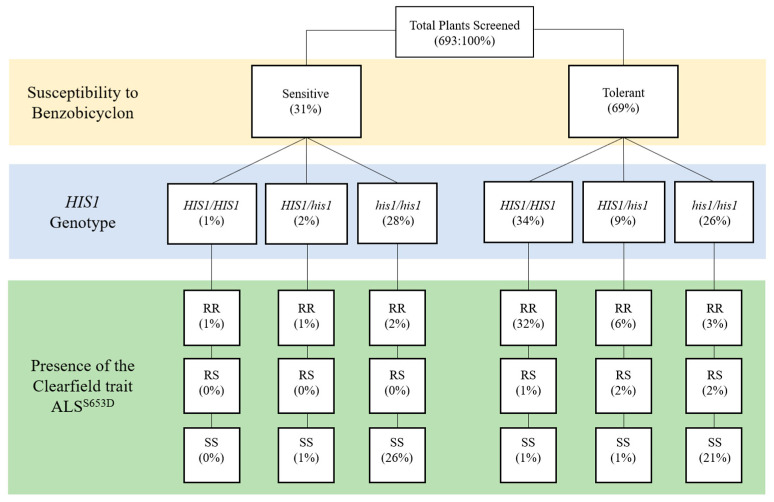
Dendogram describing the relationship between benzobicyclon susceptibility and the results from the *HIS1* and the Clearfield trait ALS^S653N^ Kompetitive Allele Specific PCR (KASP) assays. *HIS1*/*HIS1*: homozygous resistant; *HIS1*/*his1*: heterozygous; *his1*/*his1*: homozygous susceptible; RR: homozygous resistant; RS: heterozygous; SS: homozygous susceptible. Values in parenthesis are the percentage of plants with that specific genotype.

In the 19 accessions with contradicting classifications, the difference in two accessions classified as biologically sensitive, but genotyped as a *HIS1*/*his1* mixed population (accessions 18 and 23), could possibly be explained by the high level of benzobicyclon sensitivity observed in heterozygous plants. In the remaining 17 accessions, the discrepancy can primarily be attributed to the erroneous classification of plants in the KASP assay as *his1*. Fourteen of the 19 misclassified accessions were considered tolerant in the field, but classified as a *HIS1*/*his1* mixed population or sensitive (*his1*/*his1*). The other three accessions were predicted to be sensitive, but were a mixture of biologically tolerant and sensitive plants within each accession. These results can be further exemplified by comparing the plant genotype to its biological response to benzobicyclon across accessions. Not surprisingly, 98% of the 216 biologically sensitive plants were either homozygous or heterozygous for the *his1* allele; however, an additional 180 plants predicted to be homozygous for the *his1* allele were found to be tolerant to benzobicyclon (Figure 2).

### 2.3. Correlation between HIS1 and the ALS^S653N^ Clearfield Trait

In Arkansas, Clearfield-resistant *japonica* rice with the ALS^S653N^ mutation has high market penetration [19]; however, owing to gene flow, IMI-resistant weedy rice is now a widespread issue in many grower fields [8]. Interestingly, the ALS^S653N^ trait and the *HIS1* loci are localized to the lower half of chromosome two in the rice genome. Here, we are not interested in the severity of IMI-resistant weedy rice, but what is the probability the ALS^S653N^ and the *HIS1* genes co-segregate in the likely event of gene flow between weedy rice and the *japonica* cultivars grown in Arkansas. Thus, the 693 plants genotyped for *HIS1* were also genotyped for the presence or absence of the ALS^S653N^ trait using a KASP assay [7]. Figure 2 classifies plants by their biological response to benzobicyclon and their subsequent genotype for *HIS1* and ALS^S653N^. As expected, IMI-resistant weedy rice was a common genotype in 22 out of the 37 accessions (Table 1) and, in fact, 344 out of 693 plants screened contained at least one copy of the ALS^S653N^ (homozygous resistant (RR) and heterozygous (RS)) trait (Figure 2). The probability that the ALS^S653N^ trait and *HIS1* co-segregate was found to be high (ꭓ^2^ = 456, *p* < 0.0001). In the 344 plants with at least one copy of the ALS^S653N^ trait, 86% also harbored a *HIS1* allele. These results suggest that IMI resistance in weedy rice obtained via gene flow from the commonly grown *japonica* cultivars in the midsouth United States could be used as an indicator for predicting the tolerance of weedy rice to benzobicyclon.

In this study, a KASP assay was designed to have a rapid diagnostic tool to predict the sensitivity of weedy rice accessions to benzobicyclon. The assay was designed to genotype plants for the presence or absence of a fully functional *HIS1* allele normally found in *japonica* rice, but not in sensitive *indica* or *aus* cultivars. Unfortunately, the predicted *HIS1* genotype from the KASP assay did not always match the field data. For example, the KASP assay predicted 374 benzobicyclon sensitive homozygous *his1* plants, but only 52% of these were killed or significantly injured in the field. It is possible the predicted susceptible, but actually tolerant weedy rice might carry a unique resistant mechanism (e.g., point mutations in *HPPD* target gene or alternative detoxification route) or, alternatively, the heterogenous genetic makeup of weedy rice could play a role in the discrepancy with our KASP assay. Weedy rice in the midsouthern United States is believed to be derived from *indica* and *aus* subpopulations, but the genetic contribution from the wild rice progenitor, *O. rufipogon,* has contradicting reports [15,16,20]. The *HIS1* genotype of *O. rufipogon* is unknown and it is plausible that *O. rufipogon* harbors a functional *HIS1* gene that shares the SNPs used in our assay to identify benzobicyclon sensitive *indica* or *aus his1* alleles. The susceptibility of *O. rufipogon* to benzobicyclon needs to be determined to further explore this theory. Regardless, our results indicate that a new assay designed around alternative SNPs is needed to better predict the sensitivity of weedy rice to benzobicyclon. Despite the above, our field results indicate benzobicyclon alone cannot be relied upon as a viable option for weedy rice control in the midsouthern United States, which further complements the findings of Young et al. [10]. Even more troublesome, a high correlation (86%) between the prevalence of the ALS^S653N^ IMI-resistant trait and the *HIS1* gene in weedy rice was found. These findings suggest that, in fields infested with IMI-resistant weedy rice, the probability benzobicyclon will effectively kill these plants is low. However, it should be noted that benzobicyclon had considerable activity on heterozygous *HIS1* plants in accessions where the homozygous *his1* plants were sensitive in the field. Previous research has shown benzobicyclon can significantly injure transplanted *japonica* × *indica* cultivars [11,21,22,23]. For example, Kim et al. [23] found three transplanted *japonica* × *indica* cultivars, on average, exhibited 50% foliar chlorosis at 14 DAT and the tiller number per hill was reduced by one-third after treatment with benzobicyclon at 350 g ha^−1^.

Further research is needed to determine the response of homozygous and heterozygous *HIS1* plants at different growth stages in the presence and absence of crop competition, considering that previous research has shown weedy rice control can partially be a function of size at application [24,25]. Regardless, weedy rice control in non-transgenic cultivated rice will remain a difficult endeavor and an integrated weed management approach is required.

## 3. Materials and Methods

### 3.1. Plant Material

The 37 weedy rice accessions used in this study are a subsample of accessions used by Young et al. [10]. The accessions were originally collected in 2015 from rice fields predominantly in Arkansas, but also in Mississippi and Missouri. A detailed sampling methodology and collection sites (GPS coordinates) can be found therein.

### 3.2. Benzobicyclon Field Study

In the summer of 2019, a field experiment was conducted to investigate and correlate the response of weedy rice to benzobicyclon at the plant, accession, and genotypic levels. The experiment was conducted at the Milo J. Shult Agricultural Research and Extension Center in Fayetteville, AR. The previous year’s crop was soybean and the soil type was a leaf silt-loam soil (fine, mixed, active, thermic Typic albaquults) with a pH of 5.2 and 1.8% organic matter content. During the experiment, the maximum and minimum temperatures registered were 29.4 °C and 19.3 °C, respectively, with a mean temperature of 24.3 °C, and the average precipitation was 92.5 mm. The experiment was a one-factor (weedy rice accession) randomized complete block design. The entire experiment was contained in a single bay of 18 by 25 m with a 0.9 m alley separating three replications. In each block, an accession was hand planted in rows distanced 30 cm, and within a row, ten total hills (three seeds hill^−1^) were planted at 0.3 m spacings. Each accession was replicated and randomly located once within a block. The total number of plants tested for an accession ranged from 14 to 24 plants owing to unforeseen circumstances (poor germination, plants too small at application, or insufficient DNA yield after extraction). Non-treated plants were not included in this experiment, however, the *japonica* benzobicyclon-tolerant cultivar ‘LaKast’ and a benzobicyclon-sensitive *aus* cultivar ‘Purple Marker’ were included as internal tolerant and sensitive controls, respectively. Plots were fertilized and maintained weed-free using Arkansas extension recommendations for rice production [26].

Once plants reached the two-leaf stage, a hill was flagged and thinned to one plant, then at the four- to five-leaf growth stage, a 5 to 10 cm flood was established three days prior to benzobicyclon treatment and the flood was maintained for 30 DAT. At this time, plants with less than four to five leaves were pulled to remove the potential confounding effect of plant size. At application, benzobicyclon at 371 g ai ha^−1^ (Rogue^TM^, 3.3 SC, Gowan Co, Yuma, AZ, USA) plus methylated seed oil (1% *v*/*v*) was applied across the entire test area with a CO_2_-pressurised backpack sprayer with a four-nozzle handheld boom equipped with 110015 AIXR nozzles calibrated to deliver 143 L ha^−1^ at 276 kPa. At 30 DAT, plant injury was recorded on a scale from 0 to 100%, where 0 equaled a healthy plant with no visible reduction in plant growth or injury (e.g., bleaching symptomology characteristic of HPPD-inhibitors) and 100% being plant death. The response of the tolerant (LaKast) and sensitive (Purple Marker) controls were used as baselines. An accession was classified as tolerant or susceptible if ≥90% of the plants exhibited the same response.

### 3.3. DNA Extraction

Plant tissue (top third section of newly emerged leaf) of weedy rice accessions was collected at the two-leaf stage, placed in individual Eppendorf tubes, and stored at −80 °C for subsequent DNA extraction. DNA was isolated from weedy rice tissue using the CTAB (cetyl trimethylammonium bromide) protocol modified for a 96-well plate [27]. Tissue was then placed in 1.2 mL tall microtubes and frozen tissue was finely ground with two metal beads (2.4 mm diameter) on a mixer mill (Retsch, MM400) at a frequency of 30 hz s^−1^ during 15 s. Then, 300 µL of CTAB (2% CTAB, 1.4 M NaCl, 20 mM EDTA (ethylenediamine tetraacetic acid), and 100 mM Tris-HCI, pH 8.0) extraction buffer was added to each sample. DNA quality and quantity were assessed spectrophotometrically (Nanodrop 2000c, Thermo Scientific, Waltham, MA, USA).

### 3.4. KASP Assay

KASP assays were designed to detect the Clearfield ALS^S653N^ trait and *HIS1* alleles. The ALS^S653N^ KASP assay used in this study was previously created by Rosas et al. [7], and the protocol provided therein was followed. The *HIS1* KASP assay was designed to differentiate between a fully functional *HIS1* gene (e.g., *HIS1* in *japonica* cultivars) from the *HIS1* gene found in the benzobicyclon susceptible cultivars of *indica* (*his1*) and *aus* (*HIS1* *). The dataset from the 3k Rice Genome Project (http://snp-seek.irri.org [28]) was used to identify SNPs shared between *indica* and *aus* cultivars, but not found in the *HIS1* gene of *japonica*. SNPs localized in the second intron of *HIS1* at the 607th and 612th nucleotides (GAT(T/C)GATG(C/T)TTTA; tolerant/susceptible) were chosen for assay development. The SNPs of interest were sent to LGC Genomics (Beverly, MA, USA) for in-house primer design.

KASP reactions comprised a 2× KASP Master Mix, KASP Assay mix (primers), and weedy rice DNA. The reactions were run on 96-well plates on a CFX Connect Real-Time System (Bio-Rad Laboratories Inc., Hercules, CA, USA) following the directions of the manufacturer. Thermal cycle was as follows: 1 cycle of activation at 94 °C for 15 min, 10 cycles consisting of a denaturation step at 94 °C for 20 s, annealing/elongation of 61 °C (decreasing 0.6 °C per cycle) for 1 min, and 26 cycles with a denaturation step of 94 °C for 20 s and 55 °C for 1 min. Plate read was performed after a cycle at 37 °C for 1 min. Allelic discrimination was automatically assigned by the CFX Maestro software based on the relative fluorescence units of FAM and HEX fluorophores obtained at the end of the run. For the ALS^S653N^ trait assay, the cultivar LaKast was used in all reactions as a homozygous susceptible (SS) control. For the *HIS1* assay, LaKast (*HIS1/HIS1*), Purple Marker (HIS1 */HIS1 *), and a synthetic heterozygous mixture of LaKast/Purple Marker (1:1 ratio) served as homozygous tolerant, homozygous susceptible, and heterozygous standards. A no-template control where water was adding instead of DNA was included in all runs.

### 3.5. HIS1 Gene Sequencing

To verify the results from the *HIS1* KASP assay, the *HIS1* gene from three randomly selected weedy rice samples from each class (homozygous tolerant, sensitive, and heterozygous) plus LaKast and Purple Marker were sequenced. The primers used to amplify a 300 bp amplicon centered around the SNPs of interest were the forward primer 5′ATGGCAAGAACTTCCAGATTC 3′ and the reverse primer 5′AGACAACAATCCTTGTTACTTTGTAAG 3′. Each 50 µL PCR reaction consisted of 1× Colorless GoTaq Flexi Buffer (Promega Corp., WI, USA), 1.5 mM MgCl_2_, 0.2 mM dNTP’s, 0.2 µM of each forward and reverse primer, 100 ng DNA, and 1.25 units GoTaq Hot Start Polymerase. PCR conditions were as follows: 94 °C 2 min, 35 cycles of 94 °C 30 s, 51 °C 30 s, 72 °C 1 min, and a final extension of 72 °C 5 min. An aliquot of the PCR product was run on a 1.5% *w*/*v* agarose gel to assess correct amplification. PCR products were isolated using the GeneJET PCR Purification kit (Thermo Fisher Scientific, Vilnius, Lithuania) following the manufacturer’s procedures and sent to Eurofins Genomics (Louisville, KY) for Sanger sequencing. Raw sequences were compared using BioEdit [29] and Multalin [30] software.

### 3.6. Data Analysis

In order to understand the relationship between the *HIS1* genotypes and the presence of the Clearfield ALS^S653N^ traits, the observed allele frequencies of both variables were subjected to a Chi-square (ꭓ^2^) test at significance level α = 0.05. The ꭓ^2^ test was performed in JMP statistical software (SAS Institute Inc., Cary, NC, USA).

## 4. Conclusions

In this study, we have designed a KASP assay for genotyping 37 accessions of weedy rice for *HIS1* and the ALS^S653N^ traits. In addition, we have linked the susceptibility of those weedy rice accessions to benzobicyclon (a HPPD-inhibiting herbicide). We have demonstrated a significant relationship between both the *HIS1* and the ALS^S653N^ traits. Furthermore, our results suggest that the genetic origin of weedy rice would play a role in the differential susceptibility to benzobicyclon. It is likely that weedy rice with the ALS^S653N^ trait will surpass benzobicyclon effects.

## Figures and Tables

**Table 1:** Biological and genotyping designation of the 37 weedy rice accessions used in this study. Data correlate the sensitivity to benzobicyclon in weedy rice plants at field level, and the genotyping results of the *HIS1* and the ALS^S653N^ trait.

Accession	Plants Screened	Biological Data	*HIS1* Allele	ALS^S653N^ Trait
LaKast ^1^	22	Tolerant	Tolerant	Resistant
PM ^2^	20	Sensitive	Sensitive	Sensitive
8	15	Tolerant	Tolerant	Resistant
13	20	Tolerant	Tolerant	Resistant
14	22	Tolerant	Tolerant	Resistant
26	17	Tolerant	Tolerant	Resistant
33	20	Tolerant	Tolerant	Resistant
37	25	Tolerant	Tolerant	Resistant
2	15	Tolerant	Tolerant	Mixed
6	17	Tolerant	Mixed	Resistant
11	15	Tolerant	Mixed	Resistant
22	17	Tolerant	Mixed	Resistant
3	20	Tolerant	Mixed	Mixed
7	17	Tolerant	Mixed	Mixed
32	19	Tolerant	Mixed	Mixed
34	15	Tolerant	Mixed	Mixed
35	18	Tolerant	Mixed	Mixed
1	19	Tolerant	Sensitive	Sensitive
10	18	Tolerant	Sensitive	Sensitive
24	21	Tolerant	Sensitive	Sensitive
25	24	Tolerant	Sensitive	Sensitive
31	15	Tolerant	Sensitive	Sensitive
9	18	Tolerant	Sensitive	Mixed
4	22	Mixed	Mixed	Mixed
5	18	Mixed	Mixed	Mixed
27	21	Mixed	Mixed	Mixed
12	23	Mixed	Mixed	Resistant
36	15	Mixed	Mixed	Resistant
15	19	Mixed	Sensitive	Sensitive
16	14	Mixed	Sensitive	Sensitive
20	21	Mixed	Sensitive	Sensitive
17	19	Sensitive	Sensitive	Sensitive
19	18	Sensitive	Sensitive	Sensitive
21	15	Sensitive	Sensitive	Sensitive
28	18	Sensitive	Sensitive	Sensitive
29	19	Sensitive	Sensitive	Sensitive
30	19	Sensitive	Sensitive	Sensitive
23	17	Sensitive	Mixed	Resistant
18	23	Sensitive	Mixed	Sensitive

^1^ LaKast: used as internal tolerant control. ^2^ PM: Purple Marker, used as internal susceptible control.

## Supplementary Materials

The following are available online at https://www.mdpi.com/2223-7747/9/11/1576/s1, Table S1: Summary of the ALS^S653N^ trait and *HIS1* allele frequencies from 37 weedy rice accessions and their field response to benzobicyclon at 371 g ha^−1^.
