# Supplementary material for: Presence of the HPPD Inhibitor Sensitive 1 Gene and ALSS653N Mutation in Weedy Oryza sativa Sensitive to Benzobicyclon
Source: Plants (Basel). 2020 Nov 14;9(11):1576. doi: 10.3390/plants9111576 (PMC7697765; doi:10.3390/plants9111576)
Supplement: Supplementary file 1 [file plants-09-01576-s001.pdf]

Table S1. Summary of the ALS^{S653N} trait and *HIS1* allele frequencies from 37 weedy rice accessions and their field response to benzobicyclon at 371 g ha^{-1}.

Accession	Plants Screened	*HIS1* Allele			ALS^{S653N} Trait			30 DAT Control
		HIS1	HET	*his1*	R	HET	S	Avg
— # —	— # —	— # of plants –			— # of plants —			— % —
LaKast [1]	22	22	0	0	0	0	22	5
PM [2]	19	0	0	19	0	0	19	84
1	19	0	0	19	0	0	19	0
2	15	15	0	0	11	1	3	0
3	20	2	1	17	14	3	3	0
4	22	8	4	10	12	0	10	43
5	18	14	4	0	12	5	1	7
6	17	10	7	0	17	0	0	0
7	17	9	4	4	6	6	5	0
8	15	15	0	0	15	0	0	2
9	18	0	1	17	6	6	6	0
10	18	0	0	18	0	0	18	0
11	15	10	5	0	15	0	0	3
12	23	6	0	17	23	0	0	4
13	20	17	3	0	18	1	1	0
14	22	22	0	0	21	1	0	3
15	19	0	0	19	0	0	19	59
16	14	0	0	14	0	0	14	7
17	19	0	1	18	0	0	19	95
18	23	0	8	15	0	0	23	93
19	18	0	2	16	0	0	18	98
20	21	0	1	20	0	0	21	82
21	15	0	0	15	0	0	15	99
22	17	10	7	0	15	1	1	0
23	17	0	5	12	16	0	1	97
24	21	0	0	21	0	2	19	0
25	24	0	1	23	0	1	23	0
26	17	14	3	0	14	3	0	0
27	21	4	12	5	5	11	5	26
28	18	0	0	18	0	0	18	98
29	19	0	0	19	0	0	19	96
30	19	0	0	19	0	0	19	93
31	15	0	0	15	0	0	15	0
32	19	13	1	5	13	1	5	0
33	20	18	2	0	20	0	0	0
34	15	7	6	2	13	0	2	8
35	18	6	0	12	6	0	12	0
36	15	12	3	0	15	0	0	8
37	25	25	0	0	25	0	0	0

[1] LaKast: Used as internal tolerant control.

[2] PM: Purple Marker, used as internal susceptible control.
